# The efficacy of electroacupuncture for the treatment of simple female stress urinary incontinence - comparison with pelvic floor muscle training: study protocol for a multicenter randomized controlled trial

**DOI:** 10.1186/s13063-015-0560-1

**Published:** 2015-02-08

**Authors:** Tongsheng Su, Jing Zhou, Zhishun Liu, Yuelai Chen, Wei Zhang, Haoran Chu, Qiong Luo, Jin Lu, Junming An, Baoyan Liu

**Affiliations:** Shaanxi Hospital of Traditional Chinese Medicine, Xi’an, Shaanxi 710003 China; Shaanxi University of Traditional Chinese Medicine, Xianyang, Shaanxi China; China Academy of Chinese Medical Sciences, Guang’anmen Hospital, Beijing, 100053 China; Yueyang Hospital of Integrated Traditional Chinese and Western Medicine affiliated to Shanghai University of Traditional Chinese Medicine, Shanghai, 200437 China; The First Hospital of Hunan University of Chinese Medicine, Hunan, 410208 China; Anhui Hospital of Acupuncture and Moxibustion, Anhui, 230031 China; Nanjing Hospital of Traditional Chinese Medicine, Nanjing, Jiangsu 210023 China; Xi’an hospital of Traditional Chinese Medicine, Xi’an, Shaanxi 710003 China

**Keywords:** Electroacupuncture, Female stress urinary incontinence, Pelvic floor muscle training

## Abstract

**Background:**

Previous research has shown that electroacupuncture therapy has a potential therapeutic effect for simple female stress urinary incontinence. In this study, pelvic floor muscle training, the first-line treatment for stress urinary incontinence in women based on meta-analysis of numerous randomized control trials and recommended by international clinical practice, is used as a control group to demonstrate whether electroacupuncture therapy is a better method for female stress urinary incontinence.

**Methods/design:**

A randomized controlled trial has been designed to evaluate the therapeutic benefit of electroacupuncture for female stress urinary incontinence compared with pelvic floor muscle training. The safety of electroacupuncture and patient compliance will also be evaluated. Untoward reaction to the electroacupuncture, including a broken needle, fainting on acupuncture, or pain during acupuncture, will be recorded and the therapy will be stopped if an untoward reaction occurs. After we have received full ethical approval and patient consent, participants will be randomized to receive a series of 24 electroacupuncture or pelvic floor muscle training interventions. The frequency and amount of leakage will be measured as the primary outcome parameters. Secondary outcome parameters include the 1-hour pad test, the short-form of the International Consultation on Incontinence Questionnaire, patient subjective effectiveness evaluation, weekly usage of pad, and usage of specialty therapy for female stress urinary incontinence.

**Discussion:**

This trial will help to determine whether electroacupuncture is a more effective treatment than pelvic floor muscle training for patients with female stress urinary incontinence.

**Trial registration:**

ClinicalTrials.gov NCT01940432 (12 September 2013).

**Electronic supplementary material:**

The online version of this article (doi:10.1186/s13063-015-0560-1) contains supplementary material, which is available to authorized users.

## Background

**Figure 1:**
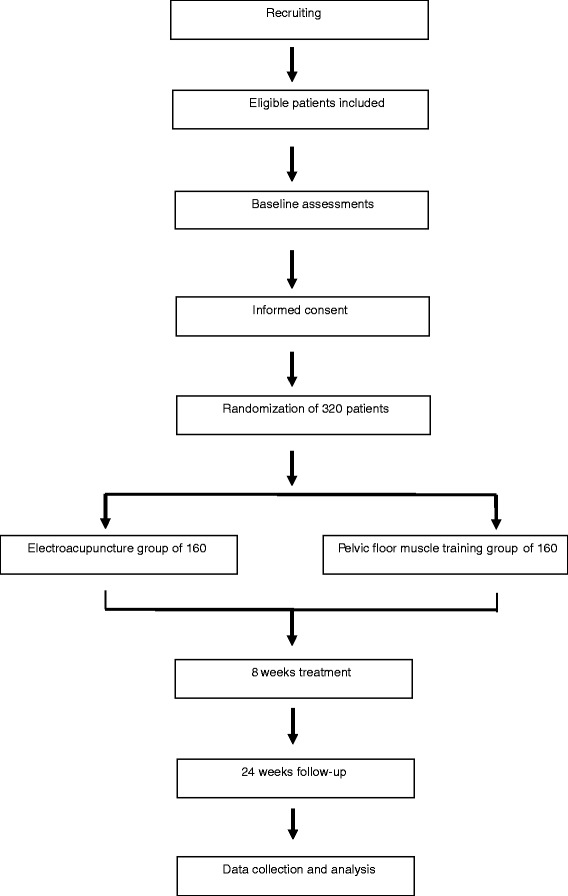
**Trial design.**

According to the International Continence Society, stress urinary incontinence (SUI) [1] is defined as involuntary loss of urine during coughing or sneezing [2]. Female SUI (FSUI) is a common condition which affects quality of life in women aged 40 to 75 years [3] (Figure 1).

In China, the prevalence of urinary incontinence in women is 46.5%, of which SUI makes up 59.6% [4]. Any women of old age are at high risk of SUI [5]. Women with SUI are embarrassed to take part in social activities, and as a result their lifestyles are altered putting them at increased risk of lifestyle-related disease, such as osteoporosis, obesity, diabetes, hypertension, coronary disease, mammary cancer, carcinoma of colon, depression, anxiety, and so forth [6].

The International Consultation on Urological Diseases recommend that treatment methods for mild and moderate SUI are lifestyle intervention, behavior therapy, pelvic floor muscle (PFM) training, functional electroacupuncture, and so forth [7]. PFM training has level A evidence of efficacy, with a curative rate ranging from 30 to 60% [8]. However, training duration should be at least 2 months. In China, PFM training is not as efficient as expected because there are not enough physical therapists, and patient compliance to treatment needs to be improved [9]. Primary therapy for moderate and severe SUI is surgery, which carries a high potential for adverse events. A safety communication from the US Food and Drug Administration on serious complications associated with transvaginal placement of a surgical mesh for pelvic organ prolapse was issued on 13 July 2011 [10]. Therefore, there is a need to explore other treatment avenues.

By contrast, acupuncture for FSUI works over a short period with a high curative rate [11], which shows potential advantages for FSUI. In a previous study [12], electroacupunture was verified as a more effective therapy for SUI than acupuncture, possibly related to the PFM and nerves. However, the level of evidence was rated as “low” [12].

A trial of high methodological quality is needed to determine whether electroacupuncture is effective for FSUI. This trial will evaluate the effect of electroacupuncture and investigate whether electroacupuncture is a better treatment for FSUI than PFM training.

## Methods/design

### Design

This study is a double-blind, multicenter, randomized controlled trial using two study arms: an electroacupuncture group and a PFM training group.

After consent, participants will be allocated to either group (the electroacupuncture group or the PFM training group) using computer generated randomization lists. Block randomization was chosen to allow for equal numbers in each group at regular time intervals. These participants will be randomly assigned to the two groups through central randomization in a 1:1 ratio. The central randomization system will be used and performed by the Clinical Evaluation Center at the China Academy of Chinese Medical Sciences in Beijing. A random number and group assignment will be immediately received by telephone, mobile phone, or website sent from the Clinical Evaluation Center. Participants will receive 24 sessions of electroacupuncture or PFM training over 8 weeks at a frequency of three times per week.

### Ethics

This study is performed in accordance with the principles of the Declaration of Helsinki [13], and has been approved by the ethics committee boards of the participating hospitals (Shaanxi Hospital of Traditional Chinese Medicine, Yueyang Hospital of Integrated Traditional Chinese and Western Medicine affiliated to Shanghai University of Traditional Chinese Medicine, The First Hospital of Hunan University of Chinese Medicine, Anhui Hospital of Acupuncture and Moxibustion, Nanjing Hospital of Traditional Chinese Medicine, Xi’an hospital of Traditional Chinese Medicine). Written informed consent will be obtained from each subject before the patient enters the trial.

### Biases

Our research is a randomized controlled trial, which is the best way to avoid selection bias, and the Clinical Evaluation Center of China Academy of Traditional Chinese Medical Sciences is responsible for the central randomization system. All of the parameters will be sealed to ensure a randomized controlled trial by removing human interference. The patient, evaluator, and statistical clerk will be blinded. Patients will be blinded to the other intervention.

Strict restrictions on inclusion criteria will be applied, and specific inclusion and exclusion criteria will restrict participants to a particular range to reduce differences between patients, making the conclusions more valid.

### Participants

Patients will be recruited and treated at the Acupuncture and Moxibustion Department in Shaanxi Hospital of Traditional Chinese Medicine and the other five co-operative hospitals of Yueyang Hospital of Integrated Traditional Chinese and Western Medicine and Shanghai University of Traditional Chinese Medicine, Anhui Hospital of Traditional Chinese Medicine, The First Hospital of Hunan University of Chinese Medicine, Nanjing Hospital of Traditional Chinese Medicine and Xi’an hospital of Traditional Chinese Medicine.

Patients will be eligible if they satisfy the following criteria:

- Aged 40 to 75 years.
- Female.
- Meet the diagnosis of simple FSUI (urine leakage associated with increased abdominal pressure from laughing, sneezing, coughing, climbing stairs, or other physical stressors on the abdominal cavity and, thus, the bladder). Also, the amount of urine loss should be greater than 1 g using the 1-hour pad test.
- Volunteered to join this research and signed the informed consent.

Potential participants will be excluded if they have any of the following:

- Urge urinary incontinence (involuntary leakage accompanied by or immediately preceded by urgency), mixed urinary incontinence (a combination of stress and urge incontinence, marked by involuntary leakage associated with urgency and also with exertion, effort, sneezing, or coughing), overflow urinary incontinence, and so forth.
- Operation for urinary incontinence or a pelvic floor operation.
- Greater than second-degree prolapse.
- Symptomatic urinary tract infection.
- Greater than 30 ml residual urinary volume.
- Less than 20 ml/second maximum urinary flow rate
- Constrained movement of walking, climbing stairs, running.
- Continuous treatment for SUI or receiving medicine for bladder function.
- Serious cardiovascular, cerebral, liver, kidney, or psychiatric disease, diabetes, Multiple System Atrophy, injury of cauda equine, myelopathy.
- Pregnancy or undergoing lactation.
- A cardiac pacemaker, metal allergy or severe needle phobia.

### Interventions

#### Electroacupuncture

The participants will be treated continuously for 8 weeks, three times per week on alternate days for 30 minutes per session, giving 24 treatments in total. The procedure is as follows. Electroacupuncture on the points Bilateral Zhong Liao (BL33) and Hui Yang (BL35) (Additional file 1) for 50 to 60 mm (using a 75-mm filiform needle). The needle will be inserted inward and downward at an angle of 30 to 45°. After insertion, the needle will be twirled, lifted and thrust three times until the patient has a needling sensation If the needle breaks during treatment it will be removed and the quality of the other needles will be checked. If a patient faints, the treatment will be suspended until they recover. If a patient feels pain, we will change the needle insertion angle and depth. The electric stimulator is applied to bilateral BL33 and BL35 with a continuous wave, 50 Hz electric current at 1 to 5 mA from the positive pole to the negative pole. The treatment design is based on literature research over the preceding 10 years, former research results and expert consensus.

#### Pelvic floor muscle training

The participants are treated continuously for 8 weeks, three times a week on alternate days, giving 24 treatments in total (Additional file 2). Before performing PFM training, patients should learn the position of the PFM. The PFM is the muscle we contract to stop passing water when urinating. Alternatively, vaginal palpation can be used to identify the specific position of the PFM. The patient can insert their finger into the vagina to feel the muscle contraction if convenient. PFM training is performed by standing/sitting/lying on the back with knees bent. Continuously contract the PFM three times, until the patient feels they cannot contract any more strongly; then hold on the contraction, and breathe twice (inhale, exhale, inhale, exhale); then relax the whole body, breathing another two times (inhale, exhale, inhale, exhale). Then contract PFM again. Repeat the above procedure for 15 minutes [14].

### Outcome measures

#### Primary outcome measures

- Difference in mean frequency of leakage over 24 hours based on a 72-hour diary (Additional file 3) compared with baseline. The mean frequency of leakage over a 24-hour period is calculated from the 72-hour voiding diaries, measured four times, in weeks 2, 4, 6 and 8. This mean frequency is compared with the baseline frequency measured at week 0.
- Difference in mean degrees of leakage over 24 hours based on a 72-hour diary, compared with the baseline. The mean quantity of fluid loss over a mean of 24 hours is calculated from the 72-hour voiding diary, measured three times, in weeks 2, 4, 6 and 8. This mean degree of leakage is compared with the baseline mean degree measured at week 0. The degree can be divided into four levels: mild degree (a very small amount of leakage with several drops); moderate degree (moderate leakage which wets the women’s underwear, but does not wet the over-trousers); and severe degree (a large amount of leakage which wet the over-trousers)
- A severity index for FSUI: frequency of leakage × amount of leakage, comparing the value of weeks 2, 4, 6 and 8 with baseline (0 week).

Scoring for frequency of leakage is defined as: 1 = less than once per month; 2 = several times per month; 3 = several times per week; 4 = every day or night.

Scoring for amount of leakage is defined as: 1 = a few drops; 2 = small splashes; 3 = more than small splashes.

Giving a severity index of: mild = 1–2; moderate = 3–6; severe = 8–9; very severe = 12. [15].

All of the primary outcome measures are based on a 72-hour diary. Every 2 weeks, patients will be asked to record the amount of fluid they drink (tea, coffee, milk, water and so forth) and leakage information (what movement they were doing when the leakage happened) for 3 consecutive days. Researchers will calculate the above primary outcome measures according to the patient’s record.

### Secondary outcome measures

- Difference in 1-hour pad test, compared with baseline. The quantity of fluid loss will be measured by 1-hour pad test, comparing the value at 8, 20 and 32 weeks with the baseline (0 week); values at 20 weeks are calculated from the total quantity of fluid loss over weeks 17 to 20 divided by 4; values at 32 weeks are calculated from the total quantity of fluid loss over weeks 29 to 32 divided by 4.
- The International Consultation on Incontinence Questionnaire-Short Form (Additional file 4) is a brief instrument used to assess the impact of urinary incontinence on patients’ lives. This is evaluated at 8, 20 and 32 weeks; values at 8 weeks are calculated by the total score at weeks 6 and 8 divided by 2.
- Patient subjective effectiveness evaluation uses three-point scoring: no help = 0; small help = 1; medium help = 2; great help = 3. This is evaluated at 8, 20 and 32 weeks; values at 8 weeks are made by the patient subjective effectiveness evaluation at week 8. If the 8-week value is not available, week 4 or week 6 can be used as the value at week 8.
- Weekly usage of pad. The value at week 8 is the average weekly usage of pads during weeks 1 to 8; the value at week 20 is the average weekly usage of pads during weeks 9 to 20; the value at week 32 is the average weekly usage of pads during weeks 21 to 32.
- Use of specialty therapy (surgery, medicine or other treatment except electroacupuncture and PFM training) for simple FSUI. The difference of use of specialty therapy for simple FSUI will be compared between the two groups during weeks 1 to 8, 9 to 20 and 21 to 32.
- Subgroup analysis: the amount of leakage with the 1-hour pad test as a measure of the curative effect of electroacupuncture for different extents of simple FSUI. Time point: 8, 20 and 32 weeks.

### Sample size

The sample size calculation was based on former study results [10]. In a previous study, the frequency of leakage using a mean 24-hour pad test in the electroacupuncture group was reduced 1.52 to 3.34 times (95% confidence intervals, and the frequency of leakage with a mean 24-hour pad test in the PFM training group was reduced 1.5 ± 1.1 times [16]. With 90% power and alpha = 0.05, and using anticipated drop-out rates of 20%, the required sample size was calculated as 320 overall.

### Data analysis

The statistical analysis will be performed by a statistician blinded to allocation in the Clinical Evaluation Center of China Academy of Chinese Medical Sciences.

Statistical analyses will be performed using the SPSS statistical package program (ver.16.0; and the level of significance will be established at <0.05. The data analysis of baseline characteristics is based on the intention-to-treat population (data of all randomized participants will be analyzed).

The data analysis of the primary and secondary outcomes is mainly based on the intention-to-treat population. In addition, the data analysis of the primary outcome is also based on the per-protocol population as a supportive analysis. For primary and secondary outcome measures, analysis of covariance will be used to investigate whether electroacupuncture is more effective than PFM training.

### Quality control

Before the trial, all staff involved will be trained on patient selection and exclusion, entering data on the case report forms and acupuncture methods. During the trial, supervisors will check on case report forms and acupuncture operation twice a month. Drop-outs, withdrawals (and the reasons) and the compliance of all patients will be recorded in detail throughout the treatment and follow-up period.

## Discussion

The results of this trial are expected to provide convincing evidence whether acupuncture is a more effective method for patients with SUI than PFM training. The trial is sponsored and financially supported by “the Twelfth Five-year Plan” from the Ministry of Science and Technology of China, which is the most important basic research program based on clinical practice.

The proposed study presents the first high quality randomized controlled trial on electroacupuncture for FSUI. As identified in a recently published review and a systematic review and meta analysis (Wang Y, Zhishun L, Peng W, Zhao J, Liu B, manuscript accepted), only a few studies have investigated electroacupuncture for FSUI [17,18]. None of these was adequately powered to allow valid conclusions on its optimal effectiveness. Additional risk of bias was introduced by methodological issues, such as invalid randomization procedures and unclear blinding, leading to a grading of the current level of evidence as “low” [10]. Therefore, we are conducting a multicenter, randomized, controlled large-scale trial to clarify whether electroacupuncture is more effective than PFM training in treating SUI.

There are two different treatment groups in this trial: the electroacupuncture group and the PFM training group. Bilateral Zhong Liao and Hui Yang are applied in the electroacupuncture group, which are regarded as the most common acupuncture prescriptions. The electric stimulator is applied to bilateral BL33 and BL35 with a continuous wave, 50 Hz, 1 to 5 mA electric current, which is expected to be the best therapeutic effect. PFM training has received Level A evidence rating in the treatment of SUI in women, based on meta-analyses of numerous randomized control trials, and is recommended in many published guidelines [19]. Thus, PFM training is used as the control group.

In this trial, we use a 1:1 randomization design comparing electroacupuncture to PFM training to evaluate the effect of electroacupuncture, and investigate whether electroacupuncture is a better treatment for SUI in comparison to PFM training. The sample size estimation ensures adequate power (90%) to allow valid conclusions. With the high prevalence of SUI and the socioeconomic burden of SUI, there is a demand for effective, safe, non-invasive and low-cost treatment options for this specific patient group. The primary outcome measures are based on authoritative literatures on comparable study interventions [20].

In conclusion, the results of this trial are expected to confirm the effectiveness of electroacupuncture and whether it is a better treatment for SUI compared with PFM training.

## Trial status

The study commenced 1 March 2014, and will be completed by 31 August 2015.

## Additional files

Additional file 1:
**Acupuncture points.**
Additional file 2:
**Pelvic floor muscle training.**
Additional file 3:
**Seventy-two-hour voiding diary.**
Additional file 4:
**International Consultation on Incontinence Questionnaire-Short Form.**

## Abbreviations

FSUI: Female stress urinary incontinence
PFM: Pelvic floor muscle
SUI: Stress urinary incontinence

## References

[1] Chen YH, Lin YN, Chen WC, Hsieh WT, Chen HY (2014). Treatment of stress urinary incontinence by ginsenoside Rh2. Am J Chin Med..

[2] Kinchen KS, Lee J, Fireman B, Hunkeler E, Nehemiah JL, Curtice TG (2007). The prevalence, burden, and treatment of urinary incontinence among women in a managed care plan. J Womens Health..

[3] Minassian VA, Drutz HP, Al-Badr A (2003). Urinary incontinence as a worldwide problem. Int J Gynecol Obstet..

[4] Yang HJY (2000). Incidence of urinary incontinence survey in Beijing. Beijing J Med..

[5] Sand PK, Owens GM, Black EJ, Anderson LH, Martinson MS (2014). Cost effectiveness of radiofrequency microremodeling for stress urinary incontinence. Int Urogynecol J..

[6] Chong EC, Khan AA, Anger JT (2011). The financial burden of stress urinary incontinence among women in the United States. Curr Urol Rep..

[7] Wood LN, Anger JT (2014). Urinary incontinence in women. BMJ..

[8] Bø K (2012). Pelvic floor muscle training in treatment of female stress urinary incontinence, pelvic organ prolapse and sexual dysfunction. World J Urol..

[9] Zhilan Y, Jianping S, Zihong S (2011). Influence factors of compliance of older women in countryside with stress urinary incontinence using pelvic floor muscle exercise treatment. Chin Gerontol J..

[10] Urogynecologic Surgical Mesh implants. [http://www.fda.gov/MedicalDevices/ProductsandMedicalProcedures/ImplantsandProsthetics/UroGynSurgicalMesh/default.htm] (accessed 21 November 2012).

[11] Mo Q, Liu Z (2013). Analysis of female stress urinary incontinence with treatment of acupuncture and moxibustion. Shang Hai J Acupunct Moxibustion.

[12] Wang Y, Zhisun L, Peng W, Zhao J, Liu B (2013). Acupuncture for stress urinary incontinence in adults. Cochrane Database Syst Rev..

[13] Idanpaan-Heikkila JE (2001). Ethical principles for the guidance of physicians in medical research - the Declaration of Helsinki. Bull World Health Organ..

[14] Incontinence Severity Index (ISI). [https://www.christianacare.org/documents/urogynecology/Incontinence-Severity-Index.pdf]

[15] Da Roza T, de Araujo MP, Viana R, Viana S, Jorge RN, Bø K (2012). Pelvic floor muscle training to improve urinary incontinence in young, nulliparous sport students: a pilot study. Int Urogynecol J..

[16] Zhu L, Lang J, Li C (2003). Development of nonsurgical methods. Chin J Obstetric Gynecol..

[17] Zhou Q, Gao W (2010). 20 Cases of female stress urinary incontinence patients with electroacupuncture. J Acupunct Moxibustion..

[18] Mo Q, Liu Z, Ma X (2013). Effective observation on stress urinary incontinence with electroacupuncture therapy. Beijing J Tradit Chin Med..

[19] Dumoulin C, Glazener C, Jenkins D (2011). Determining the optimal pelvic floor muscle training regimen for women with stress urinary incontinence. Neurourol Urodyn..

[20] Sandvik H, Seim A, Vanvik A, Hunskaar S (2000). A severity index for epidemiological surveys of female urinary incontinence: comparison with 48-hour pad-weighing tests. Neurourol Urodyn..

