# Supplementary material for: The efficacy of electroacupuncture for the treatment of simple female stress urinary incontinence - comparison with pelvic floor muscle training: study protocol for a multicenter randomized controlled trial
Source: Trials. 2015 Feb 8;16:45. doi: 10.1186/s13063-015-0560-1 (PMC4336724; doi:10.1186/s13063-015-0560-1)
Supplement: Additional file 1: — Acupuncture points. [file 13063_2015_560_MOESM1_ESM.docx]

| Points | Method of needling | Electric stimulator |
| --- | --- | --- |
| Bilateral Zhong Liao (BL33)  (located on  the lumbosacral region) | are given acupuncture of 50-60mm with 30-45° angle to inward and downward by 75mm filiform needle. Twirl, lift and thrust for 3 times, until local sour and heavy feeling coming. | The electric stimulator is applied to bilateral BL33 and BL35 with continuous wave, 50 Hz and electric current 1-5 mA (milliampere). |
| Bilateral Hui Yang (BL35)  (located on  the lumbosacral region) |  |  |
