# Supplementary material for: The efficacy of electroacupuncture for the treatment of simple female stress urinary incontinence - comparison with pelvic floor muscle training: study protocol for a multicenter randomized controlled trial
Source: Trials. 2015 Feb 8;16:45. doi: 10.1186/s13063-015-0560-1 (PMC4336724; doi:10.1186/s13063-015-0560-1)
Supplement: Additional file 2: — Pelvic floor muscle training. [file 13063_2015_560_MOESM2_ESM.docx]

**Pelvic Floor Muscle Training Guidance**

| **Examples** | **Guidance** | **Position** | **Notes** |
| --- | --- | --- | --- |
| 1 | identify anal sphincter/feel anus | sitting | try to raise it from chair (without adding abdominal, thigh, and buttock muscles) |
| 2 | identify levator ani | sitting, bent forward, elbows on knees | try to raise vagina from chair (without adding abdominal, thigh, and buttock muscles) |
| 3 | contract levator ani | sitting, lying and standing | it feels like elevator raising up, with a 10s break between contractions, which can be used vaginal palpation to identify |
| 4 | contract anal sphincter | sitting, lying and standing. | it feels like elevator raising up, with a 10s break between contractions, which can be used vaginal palpation to identify |
