# Supplementary material for: The efficacy of electroacupuncture for the treatment of simple female stress urinary incontinence - comparison with pelvic floor muscle training: study protocol for a multicenter randomized controlled trial
Source: Trials. 2015 Feb 8;16:45. doi: 10.1186/s13063-015-0560-1 (PMC4336724; doi:10.1186/s13063-015-0560-1)
Supplement: Additional file 3: — Seventy-two-hour voiding diary. [file 13063_2015_560_MOESM3_ESM.doc]

**排 尿 日 记 卡**

**第|__|周**

**（201|__|年|__|__|月|__|__|日-201|__|年|__|__|月|__|__|日）**

| **入组序号: |__|__|__|** |
| --- |
| **姓 名：____________**  **电 话：____________** |

**填写排尿日记卡注意事项**

1. 本日记卡能够很好地反映您的病情变化，请您认真及时填写。您填写的内容对进一步治疗有很大帮助，请避免缺项、漏项或错填。感谢您的配合！
2. 从进入研究的第一天起，直至研究结束，整个34周研究期间原则上**不允许使用治疗压力性尿失禁的药物和其他专科治疗**（主要指生物反馈疗法、盆底阴道或肛门电刺激或磁刺激疗法、盆底经皮电刺激等）。若必须使用，请在泌尿科医师指导下用药，并在“**压力性尿失禁专科治疗和尿垫使用情况记录表**”中做记录**。**
3. 第4天至第6天需填写72h排尿日记，请根据示例认真规范的记录各项内容。在此3天内，每次饮水时，请使用课题组配备的带刻度水杯计算饮水量。
4. 尿失禁栏中的“状态”指发生尿失禁时您正在做什么，比如跑步、咳嗽、赶公交车等，请认真记录。
5. 饮水类型：指水、牛奶、茶、咖啡、碳酸饮料、酒等。
6. 若您需使用尿垫，请在排尿日记卡“今日尿垫用量”处记录尿垫的使用个数。
7. 若您在填写排尿日记卡期间患有较重的咳嗽，并因此增加了尿失禁次数和漏尿量，请在“较重的咳嗽”处勾选“是”或“否”。
8. 排尿日记卡最后一页“72h排尿日记卡汇总”的内容由医生填写。

**排尿日记卡**

**201|__|年|__|__|月|__|__|日**

| **尿失禁** | **饮水** |
| --- | --- |
| 时间/次数/状态^*^ | 时间/类型/饮水量（ml） |
| **早6:00** | **早6:00** |
| 6:23 1次 下床 | 6:50 1杯水 100ml |
|  |  |
|  |  |
|  |  |
|  |  |
| **中午12:00** | **中午12:00** |
|  |  |
|  |  |
|  |  |
|  |  |
| **下午18:00** | **下午18:00** |
|  |  |
|  |  |
|  |  |
|  |  |
| **午夜12:00** | **午夜12:00** |
|  |  |
|  |  |
|  |  |
|  |  |
| **请您填写：**今日尿垫用量：|__| **个；** 较重的咳嗽：**□ 是 □ 否** | |

**排尿日记卡**

**201|__|年|__|__|月|__|__|日**

| **尿失禁** | **饮水** |
| --- | --- |
| 时间/次数/状态^*^ | 时间/类型/饮水量（ml） |
| **早6:00** | **早6:00** |
| 6:23 1次 下床 | 6:50 1杯水 100ml |
|  |  |
|  |  |
|  |  |
|  |  |
| **中午12:00** | **中午12:00** |
|  |  |
|  |  |
|  |  |
|  |  |
| **下午18:00** | **下午18:00** |
|  |  |
|  |  |
|  |  |
|  |  |
| **午夜12:00** | **午夜12:00** |
|  |  |
|  |  |
|  |  |
|  |  |
| **请您填写：**今日尿垫用量：|__| **个；** 较重的咳嗽：**□ 是 □ 否** | |

**排尿日记卡**

**201|__|年|__|__|月|__|__|日**

| **尿失禁** | **饮水** |
| --- | --- |
| 时间/次数/状态^*^ | 时间/类型/饮水量（ml） |
| **早6:00** | **早6:00** |
| 6:23 1次 下床 | 6:50 1杯水 100ml |
|  |  |
|  |  |
|  |  |
|  |  |
| **中午12:00** | **中午12:00** |
|  |  |
|  |  |
|  |  |
|  |  |
| **下午18:00** | **下午18:00** |
|  |  |
|  |  |
|  |  |
|  |  |
| **午夜12:00** | **午夜12:00** |
|  |  |
|  |  |
|  |  |
|  |  |
| **请您填写：**今日尿垫用量：|__| **个；** 较重的咳嗽：**□ 是 □ 否** | |

**72h排尿日记卡汇总**

**以下资料由医生填写：**

| **1. 平均24h尿失禁次数：** **次**  计算方法：72h内尿失禁次数总和除以3。 |
| --- |
| **2. 平均24h尿失禁漏尿程度：**□Ⅰ轻度漏尿 □Ⅱ中度漏尿 □Ⅲ重度漏尿  Ⅰ（轻度漏尿）：指少量漏尿， 仅漏几滴；尿垫未见成片浸湿（若使用）；  Ⅱ（中度漏尿）：指中等量漏尿，漏尿较多，可湿透内裤，但外裤未成片浸湿；可见尿垫成片浸湿（若使用）；  Ⅲ（重度漏尿）：指大量漏尿，漏尿多，不仅内裤湿透，同时外裤也成片浸湿；一次漏尿即可将尿垫成片浸湿（若使用）。 |
| **3. 72h尿垫用量：** **个**； |
| **4. 平均24h饮水量：** ml |
| **4. 较重的咳嗽：□ 是 □ 否** |
