# Supplementary material for: The efficacy of electroacupuncture for the treatment of simple female stress urinary incontinence - comparison with pelvic floor muscle training: study protocol for a multicenter randomized controlled trial
Source: Trials. 2015 Feb 8;16:45. doi: 10.1186/s13063-015-0560-1 (PMC4336724; doi:10.1186/s13063-015-0560-1)
Supplement: Additional file 4: — International Consultation on Incontinence Questionnaire-Short Form. [file 13063_2015_560_MOESM4_ESM.docx]

**国际尿失禁咨询委员会尿失禁问卷表简表（ICIQ-SF）**

许多患者时常溢尿，该表将用于调查尿失禁的发生率和尿失禁对患者的影响程度。仔细回想你近四周来的症状，尽可能回答以下问题。

1．您的出生日期： |___|____|____|___|年|___|___|月|___|___|日

2．性别（在空格处打√） 男 □ 女 □

| 3．您漏尿的次数？  （在一空格内打√）  从来不漏尿□ 0  一星期大约漏尿1次或经常不到1次□ 1  一星期漏尿2次或3次□ 2  每天大约漏尿1次□ 3  一天漏尿数次□ 4  一直漏尿□ 5 |
| --- |
| 4． 我们想知道您认为自己漏尿的量是多少？  在通常情况下，您的漏尿量是多少（不管您是否使用了防护用品）  （在一空格内打√）  不漏尿□ 0  少量漏尿□ 2  中等量漏尿□ 4  大量漏尿□ 6 |
| 5．总体上看，漏尿对您日常生活影响程度如何？  请在0（表示没有影响）~ 10（表示有很大影响）之间的某个数字上画圈  0 1 2 3 4 5 6 7 8 9 10  没有影响 有很大影响 |

ICIQ-SF评分（把第3、4、5个问题的分数相加）：|___|___|

| 6.什么时候发生漏尿？  （请在与您情况相符合的那些空格内打√）  从不漏尿□  未能到达厕所就会有尿液漏出□  在咳嗽或打喷嚏时漏尿□  在睡着时漏尿□  在活动或体育运动时漏尿□  在小便完和穿好衣服时漏尿□  在没有明显理由的情况下漏尿□  在所有时间内漏尿□ |
| --- |

非常感谢您回答以上的问题！
